# Complex trigger wrist associated with carpal tunnel syndrome: a case report

**DOI:** 10.1186/s13256-022-03645-8

**Published:** 2022-11-13

**Authors:** Alexey V. Lychagin, Dmitry S. Bobrov, Kirill D. Artemov, Yaroslav A. Rukin, Vadim G. Cherepanov, Ekaterina E. Rudenko, Andrey B. Ponomarev

**Affiliations:** 1 The Department of Traumatology, Orthopedics and Disaster Surgery, Sechenov University, Botkin Hospital, Bolshaya Pirogovskaya 6, B. 1, 119435 Moscow, Russian Federation; 2 The Department of Traumatology, Orthopedics and Disaster Surgery, Sechenov University, 8-2 Trubetskaya Str, Moscow, Russian Federation; 3 Institute of Clinical Morphology and Digital Pathology, Sechenov University, 8-2 Trubetskaya Str, Moscow, Russian Federation

**Keywords:** Wrist, Finger, Trigger, Carpal tunnel

## Abstract

**Background:**

There are few reports of trigger wrist in the literature, as it is a rare pathology. Furthermore, various authors report that it is also hard to diagnose. It manifests with neurological symptoms at the affected wrist, which are usually induced by wrist movement, and can lead to partial or full loss of wrist function and sensitivity. The reason for reporting this specific case is that it was hard to differentiate between trigger finger and trigger wrist by clinical symptoms; no pathology was palpable or clearly seen on magnetic resonance imaging scan of the wrist. We propose a new diagnostic statement relative to this pathology.

**Case presentation:**

A case of a 45-year-old white slavic man with trigger wrist associated with carpal tunnel syndrome, caused by a fibroma of the flexor tendon sheath, is reported. Despite careful clinical examination, it was not possible to differentiate between trigger finger and trigger wrist. Magnetic resonance imaging was performed to arrive at the right diagnosis but did not reveal any pathology in the wrist area. Carpal tunnel release was performed with a fibroma identified and excised. Wrist function was maintained well; no signs of carpal tunnel syndrome were seen at last follow-up.

**Conclusions:**

Trigger wrist can be misdiagnosed as trigger finger even if adequate clinical evaluation is performed, and this can lead to inadequate treatment. We state that, when clinical symptoms of both trigger wrist and trigger finger are present, except painful palpation of the A-1 pulley region, the case should be referred to as trigger wrist.

## Background

Trigger wrist is a rare pathology that can lead to loss of wrist function [1, 2, 4]. It is defined as an occurrence of triggering or painful clicking at the wrist joint level, induced by wrist or finger movement [2, 4]. Such symptoms are caused by temporary compression of the nerves in the carpal tunnel or extensor retinaculum area by tumor or adhesions [4]. The most frequent reason for this condition is fibroma of the flexor tendons or tendon sheath [1, 4]. We report a clinical case of a patient with complex trigger wrist caused by fibroma of the flexor tendon sheath. We believe this case to be complex and unique because of the difficult differential diagnosis between trigger finger and trigger wrist and unclear results of clinical examination. We also present a literature review and demonstrate the classifications used for this pathology.

## Case presentation

**Fig. 1:**
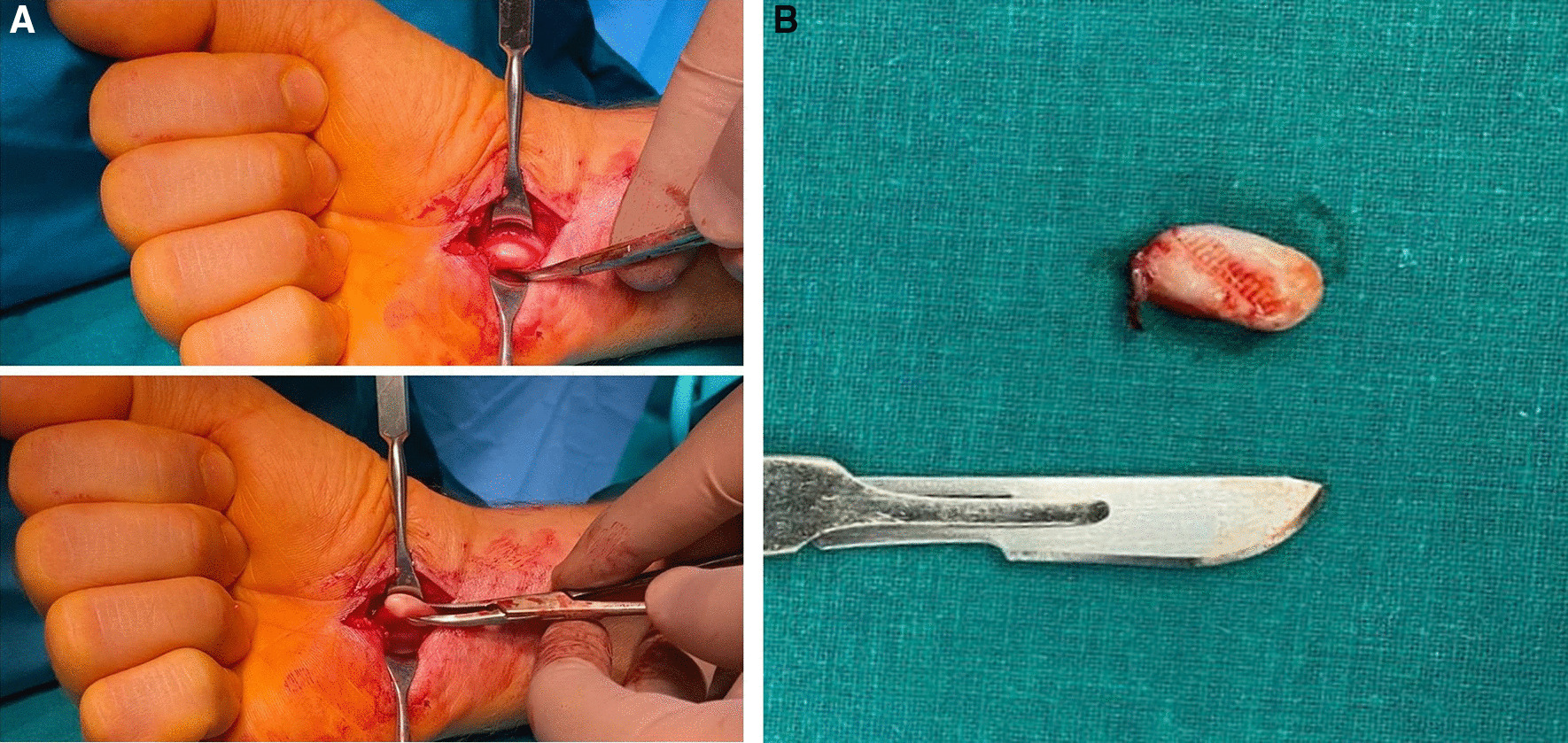
**a** The release of the carpal tunnel. Oval-shaped fibroma identified inside the carpal tunnel. **b** Fibroma after excision. The size of the tumor is 1 × 2.5 cm^2^

**Fig. 2:**
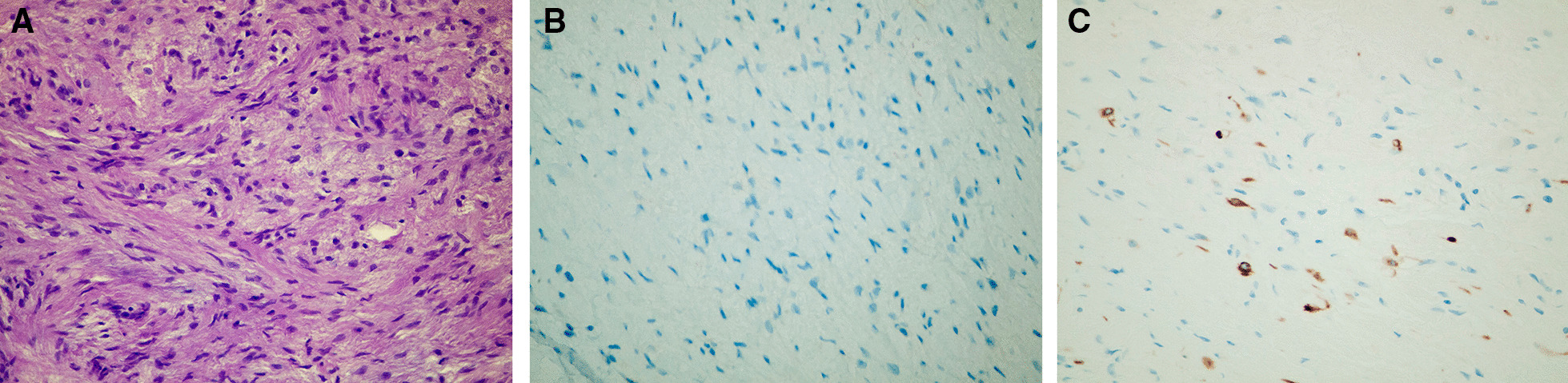
**a** Histological examination, hematoxylin and eosin (H&E), 40×: the tumor node is formed by spindle-shaped or oval-shaped cells with rather large nuclei of an elongated, rounded, or polygonal shape. Cells are located loosely in places; in places they form compact clusters. Some nuclei show one or two nucleoli. A morphological picture of the fibroma. **b** For immunohistochemical analysis, S100 and desmin were used as primary antibodies. S100 was used for differential diagnosis with peripheral nerve tumors—negative immunoperoxidase reaction, 40×. **c** Focal cytoplasmic expression of desmin was seen after immunoperoxidase reaction, 40×;—it is characteristic of cells with a myoid phenotype and it can be detected in 50% of cases of fibromatosis. This tumor in the development of relapse can also be attributed to fibromatosis, given its localization

A 45-year-old white slavic male presented with 4-year history of “clicking” of the right wrist. For the last 2 years, pain at the right wrist joint level and fourth finger flexion failure had also occurred. All the symptoms were induced by wrist and fourth finger flexion and extension. Recently, the patient had noted weakening of the right wrist. Examination did not reveal any visible or palpable pathology at the wrist level. Weakening of the right wrist was noted while the patient was grabbing the examiners’ hand. Neurological examination revealed reduced sensitivity (pain) via the median nerve area at the right wrist. Triggering at the right wrist was noted during flexion and extension of the radiocarpal joint. Despite the obvious presence of carpal tunnel syndrome, Tinel’s sign was not present. MRI of the right wrist was performed: no pathological masses were seen inside the carpal tunnel. The compression of the right median nerve was confirmed by electroneuromyography. Carpal tunnel release was performed. An oval-shaped fibroma was identified in the carpal tunnel (Fig. 1a). The tumor was attached to the flexor superficialis tendon sheath and was moving through the carpal tunnel via fourth finger and wrist flexion and extension. The fibroma was excised, and the wound was closed (Fig. 1b). A morphological study was performed (Fig. 2a). Immunohistochemical analysis was performed for differential diagnosis with peripheral nerve tumors (Fig. 2b). The tumor was composed of cells with a myoid phenotype (Fig. 2c). The postoperative protocol included positioning the hand in a resting splint, regular wound dressings, and antibiotics for 3 days. All fingers were left free for regular active finger exercises. The patient was discharged after 3 days of the postoperative period. Sutures were removed after 2 weeks after surgery. The patient was followed up at 2, 4, and 6 weeks after the operation. During the last follow-up visit, there were no signs of trigger wrist or carpal tunnel syndrome.

## Discussion and conclusions

Several classifications can be found in literature. Historically, the first classification of trigger wrist was offered by Suematsu *et al.* (Table 1) [1]. It is based on etiological factors. Various authors report that this classification is not informative enough [2, 3, 6, 7]. Al-Qattan *et al.* (Table 2) [3] offered an extended classification. The reported case is classified as type A, which is the most common type of trigger wrist [1–3].

**Table 1:** Classification of trigger wrist by Suematsu *et al.* [1]

Type	Etiology
A	Tumor (occurs on the flexor tendon (sheath) enters and leaves the carpal tunnel)
B	Anomalous muscle belly (lumbrical/f. superficialis enters and leaves the carpal tunnel)
C	Combination of types A & B

**Table 2:** Classification of trigger wrist by Al-Qattan *et al.* [3]

Type	Etiology
A	Tumor or inflammatory mass that may originate from the median nerve, flexor tendon, or other contents of the carpal tunnel
B	Anomalous muscle crossing the carpal tunnel
C	Tumor within an anomalous muscle crossing the carpal tunnel
D	Partial flexor or extensor tendon injury at the wrist level
E	Mechanical causes such as snapping dislocation of a flexor tendon over the hook of the hamate (may occur after carpal tunnel release), and tendon adhesions between the flexor pollicis longus and other flexor tendons within the carpal tunnel (may occur following surgery within or near the carpal tunnel)

Differential diagnosis of trigger wrist and trigger finger is important [4, 5]. Trigger finger, also known as stenosing tenosynovitis, is usually caused by a size mismatch between the first annular pulley (A-1) and the flexor tendon [5]. More rarely it can be caused by a pathology inside the carpal tunnel [9]. Unlike trigger wrist, trigger finger is a common disease [5–8]. The symptoms of trigger finger include clicking, snatching, or triggering in the finger area, induced by the finger movement [4, 5, 8]. Clinically, while palpating the A-1 area, pain can be present [5]. In the reported case, the patient expressed no pain around the A-1 area, so the presence of stenosing tenosynovitis was unlikely. However, if trigger finger caused by stenosing tenosynovitis is mistaken for trigger wrist, then this can lead to an unnecessary release of the carpal tunnel, which introduces the risk of damaging its structures and development of adhesions [4, 8]. The adhesions within the carpal tunnel can be an etiology of trigger wrist type E by Al-Qattan *et al.* [3]. Another reason for the development of the adhesions is rheumatoid arthritis [6, 7]. Giannikas *et al.* [6] and Minetti *et al.* [7] reported several cases of trigger wrist induced by adhesions of flexor tendons inside the carpal tunnel, caused by rheumatoid arthritis.

Park *et al.* [4] also reported cases of trigger wrist with difficult diagnosis: clinically the condition was referred to as trigger finger, although there was a palpable mass inside the carpal tunnel and clicking occurred around the wrist joint area. It is stated that augmented CT scan was not informative enough [4]. The diagnosis of trigger wrist was finally stated when the carpal tunnel was revised and an anomalous muscle belly was found [4].

In our patient, carpal tunnel syndrome was present, but Tinel’s sign was not revealed—this phenomenon was also demonstrated by Macdonald *et al.* [10]. The authors present a trigger finger case caused by a ganglion, located inside the carpal tunnel, with associated carpal tunnel syndrome and negative Tinel’s sign [10].

Similar cases demonstrating that trigger finger is not always caused by A-1 pulley pathology can be found: Parmaksızoğlu *et al.* [9] reported three patients with trigger finger at the carpal tunnel level with the A-1 pulley intact. All of them were caused by a tumor inside the carpal tunnel [9].

In our patient, clicking and pain were located mainly around the wrist joint area, which was in favor of trigger wrist diagnosis. However, the fact of the fourth finger “locking” and other symptoms induced not only by wrist movement but also by fourth finger movement were the main factors to consider this case complex in terms of differential diagnosis between trigger finger and trigger wrist. Furthermore, the fibroma in the reported patient was not palpable and was not seen clearly on the MRI scan. We state that, in a case where clinical symptoms of both trigger wrist and trigger finger are present, except painful palpation of the A-1 pulley region, the diagnosis should be considered as trigger wrist. Pain around the A-1 area makes the diagnosis of trigger wrist less possible. Further research is needed.

A case of trigger wrist with a difficult differential diagnosis is presented. Despite the complexity of the case, a correct surgical procedure was performed. All the symptoms regressed, and the patient was satisfied with the treatment.

## Data Availability

The dataset supporting the conclusions of this article is included within the article.
